# Impacts of COVID-19 on people and sea: marine social science imaginations

**DOI:** 10.1007/s40152-022-00270-5

**Published:** 2022-05-18

**Authors:** Per Knutsson, Maarten Bavinck

**Affiliations:** 1 School of Global Studies, University of Gothenburg, P.O. Box 700, 405 30 Göteborg, Sweden; 2 Faculty of Social and Behavioural Sciences, University of Amsterdam, Nieuwe Achtergracht 166, Postbus 15629, 1001 Amsterdam, NC Netherlands

**Keywords:** Marine social sciences, COVID-19, Marine sector implications

It is now more than 2 years that the COVID-19 crisis — which includes the pandemic as such but also the often drastic policy measures intended to curb it — began to unfold, profoundly affecting human lives along coastlines and in maritime regions (as well as elsewhere). In line with many other scholars, marine social scientists have come forward to document how the lives of fishers, oil platform workers, tourist sector workers, marine spatial planners, and many others have been profoundly altered in various country settings.

In the early moment of challenge, the editors of *Maritime Studies* requested marine social scientists of all disciplines to submit papers enquiring into the impacts of COVID-19 on the realm of “People and the Sea.” This invitation followed from the *Manifesto for the marine social sciences* (Bavinck and Verrips 2020) that the community of social scientists gathered in the MARE Conference (2019) had formulated. Recognizing that oceanic and coastal environments and their human dimensions differ substantially from terrestrial ones, the Manifesto called for critical enquiry and reflection on the specific circumstances of coastal peoples and their futures. In addition to empirical findings, the call therefore asked potential authors to submit “marine social science imaginations of Covid-19.”

High-level UN initiatives such as the 2030 Agenda for Sustainable Development, the 2017 and 2022 UN Ocean conferences, and the UN Decade of Ocean Science for Sustainable development have put the international spotlight on the tremendous importance of the ocean for the present and future wellbeing of humans across the world. The relationships between humans and the ocean are, however, recognized to be extremely diverse (Allison et al. 2020). For supporting and improving human wellbeing and realizing a sustainable and inclusive ocean economy, policymakers are therefore urged to recognize and build upon diversity. In the wake of international debates, a growing community of social scientists, committed to the study of a broad range of aspects of human-sea relations, have stressed the urgency of a marine social science lens in addressing pressing ocean sustainability challenges (e.g., Bennett 2019; Arbo et al. 2018).

While the full scope of the pandemic still remains opaque, it has become painstakingly clear that groups and sectors depending on ocean and coastal resources have been among the hardest hit by measures imposed to contain the spread of COVID-19 (Verchuur, Koks & Hall 2021; Bennett et al. 2020; Gössling, Scott & Hall 2020; Belton et al. 2020). Taking a longer-term perspective on the policy implications of the pandemic, marine social scientists has taken different routes. While some have ventured a more practical, adaptive approach (see, e.g., Perillo et al. 2021), others point out that, in line with arguments made in wider circles (see, e.g., Gupta et al. 2021; Büscher et al. 2021), the pandemic necessitates and provides opportunities for a wider societal reset.

Responses to the pandemic thus re-affirms the *Manifesto*’s call for an inclusion of critical as well as solution-oriented marine social science perspectives into ocean sustainability and blue economy agendas. As we see it, the twelve contributions in this special issue — which belong to what one might call the second wave of COVID-19 scholarship — direct the spotlight on a range of important marine social science concerns. They do so in a manner that is distinctive from the early generation of scholarship, which predominantly hurried to document what was happening on the ground. The second generation has a more profound purpose than documentation alone: it compares across geographical regions and offers both theoretical and practical reflections.

The special issue gathers unique insights into the new marine world of the ongoing COVID pandemic across a broad diversity of contexts and sectors in Africa, Asia, and South and North America, as well as Europe. While more or less all of the contributions showcase dramatic consequences of the health crisis on blue activities and sectors, three of them provide in-depth explorations of how oceanic activities have been affected by the challenges of restrictions imposed to contain the spread of the virus. Sackey et al. (2021) investigate the challenge of making trade-offs between the need for on-site marine warranty surveys in relation to offshore marine operations in Africa on the one hand, and adhering to COVID-19 restrictions and protocols on the other. While trade-offs are apparently unavoidable, the authors also note the emergence of innovative socio-technical approaches to manage them. Bringing us to the context of the Mexican Caribbean, Fraga et al. (2022) probe the extraordinary risk posed by the “double-impact” of coastal *Sargassum* algal blooms and a 98% contraction of the tourist industry during 2020 due to harsh COVID-19 containment measures. Drawing on Ulrich Beck’s concept of “risk society,” they bring forward narratives from workers within both the tourist and fisheries sector that provide unique insights into a society that is forced to transform in response to complex, man-made uncertainties. Köpsel et al. (2021), on the other hand, take a look at EU marine science projects and explore how the requisite of stakeholder participation and engagement has been affected by lockdowns and social distancing demands. Learning from the experiences in a number of ongoing projects, the study suggests practical solutions to sustain stakeholder engagement in times when physical social relations are restricted.

Several of the contributions included in the special issue demonstrate how the extraordinary consequences of the COVID-19 pandemic can be used as a lens through which existing structures and dynamics of vulnerability, as well as the set of obstacles and opportunities for adaptive responses, become acutely visible. Bassett et al. (2022), Manlosa et al. (2021), Khan et al. (2021), Marschke et al. (2021), and Lam (2021) all probe vulnerabilities in relation to marine food systems and supply chains. Bassett et al. (2022) examine the disruption of fishery supply chains by the pandemic to comparatively assess the vulnerability and resilience of small-scale fisheries supply chains in diverse settings of Indonesia, Philippines, Canada, and the USA. Zeroing in on the context of aquaculture and capture fisheries production and marketing in the Philippines, Manlosa et al. (2021) share important insights on people’s coping strategies, and the factors that enables them. They highlight the expansion of practices such as door-to-door peddling that were marginal before and the importance of social support such as food aid.

In a similar vein, Viteri Mejía et al. (2020) effectively showcase that while the crisis caused by COVID-19 threatened the local economy in the Galapagos islands to collapse by fundamentally altering the conditions for livelihoods and food security, artisanal fishers developed strategies and initiatives by shifting roles from supplying marine food products to the tourist industry to local households. The authors argue that this shift was not only important in terms of ensuring a continued economic viability of artisanal fisheries and local food security, but also altered local perceptions of fishers and fisheries: from traditional notions of fisheries as anti-conservation and commonly being in conflict with the tourist industry, to fisheries as protagonists of the importance of marine ecosystem services and resilient suppliers of fresh, high-quality protein to local consumers.

Drawing on attempts to strengthen local aquatic food production systems in Africa, Khan et al. (2021) note that the pandemic raises urgent queries about how fish supply chains could assist in reducing malnutrition and gender inequality. They argue in favor of a comprehensive blue economy investment plan for aquatic food systems in the continent that brings about nutritional well-being, livelihoods and social inclusion, intra-regional trade, and resilient economies.

Marschke et al. (2021) focus on COVID-19 impacts on one category of actors in supply chains, namely the migrant fishworkers in Thailand and Taiwan. They connect this to the ongoing debate on unacceptable work conditions in industrial fisheries, highlighting three themes: (1) employment disruptions, (2) mobility restrictions, and (3) poor access to social services.

Taking a distinctly different perspective, Lam (2021) proposes that COVID-19 has opened up a unique window for transforming the sustainability and ethics of the global seafood industry. Through a conceptualization of how seafood industry actors’ diverse values and identities are made salient by both scale and context, the author provides empirical examples from India, Canada, and New Zeeland in a plea for ethical governance frameworks that integrates diverse values and identities.

Resonating with Lam’s emphasis on both scale and context, Lamers and Student (2021) provides an engagement with the simultaneously globalized and localized tourist industry. They propose that the ongoing pandemic offers a unique opportunity to understand the positive and negative environmental implications of tourist destinations during a time period without tourist flows.

Two of the special-issue contributions take the task of imagining marine futures in times of high uncertainty seriously. Taking the uncertainty entailed by “the known unknowns” of the COVID-19 pandemic as a conceptual starting point, Heazle (2021) presents an assessment of the pandemic’s long-term influence on marine plastic pollution and overfishing in the South China Sea in terms of undermining environmental protection and regulatory efforts. van Tatenhove (2021) instead imagines the re-development of EU marine sectors when the pandemic finally is over. By outlining four potential maritime futures that expresses different answers to fundamental questions about the future EU political and economic pathways, van Tatenhove points at both enabling and constraining conditions to deal with the COVID-19 aftermath.

Following up on the United Nations Declaration on the Rights of Indigenous Peoples (2007) and related discussions, Blanco-Wells et al. (2021) showcase how a global health crisis can contribute to opening up new possibilities. Their study interrogates the effects of the pandemic on the indigenous Yagan people in Southern Chile and bring into view how pandemic risks have come to form part of a broader sociohistorical debate on the rights of coastal peoples to their maritories. They suggest that the pandemic has thus stimulated an emancipatory ethnic revitalization of Yagan identities.

The final paper in this special issue is unique in its presentation on the COVID-19 crisis in Sri Lanka as “an early foray into the poetics and politics of viral contagion and the (un)making of diverse forms of ‘islandness’” (Godamunne et al. 2022). The authors point to continuities in the Sri Lankan history of medical quarantine but also to how the war-time (1983–2009) experience of securitization has put a mark on the style of lockdown management. Seafood production and trade — as boundary activities — were simultaneously defined as a particularly risky activity and physically cordoned off; meanwhile, the authorities symbolically “cured” the island of COVID-19 in a manner reminiscent of historical myths in which the island was cleansed of foreign invaders.

## References

[CR1] Allison, E.H., J. Kurien, Y. Ota et al. 2020. *The human relationship with our ocean planet*. Washington, DC: World Resources Institute. https://oceanpanel.org/blue-papers/ HumanRelationshipwithOurOceanPlanet

[CR2] Arbo, P., Knol, M., Linke, S et al. 2018. The transformation of the oceans and the future of marine social science. *Maritime Studies* 17: 295–304. 10.1007/s40152-018-0117-5.

[CR3] Bassett HR, Sharan S, Suri SK (2022). A comparative study of small-scale fishery supply chains’ vulnerability and resilience to COVID-19. Maritime Studies.

[CR4] Bavinck, M., Verrips, J. 2020. Manifesto for the marine social sciences. *Maritime Studies* 19: 121–123. 10.1007/s40152-020-00179-x.

[CR5] Belton, B., L. Rosen, L. Middleton et al. 2021. COVID-19 impacts and adaptations in Asia and Africa’s aquatic food value chains. Marine Policy 129 10.1016/j.marpol.2021.104523 10.1016/j.marpol.2021.104523 PMC8564473 34744258

[CR6] Bennett, N. J. 2019. Marine Social Science for the Peopled Seas. *Coastal Management *47: 2, 244–252. 10.1080/08920753.2019.1564958.

[CR7] Blanco-Wells G, Libuy M, Harambour A (2021). Plagues, past, and futures for the Yagan canoe people of Cape Horn, southern Chile. Maritime Studies.

[CR8] Büscher B, Feola G, Fischer A et al. 2021. Planning for a world beyond COVID-19: Five pillars for post-neoliberal development. *World Development* 140: 105357. 10.1016/j.worlddev.2020.105357. 10.1016/j.worlddev.2020.105357 PMC8446704 34548744

[CR9] Büscher, B., G. Feola, A. Fischer et al. 2020. Planning for a world beyond COVID-19: five pillars for post-neoliberal development, *World Development* 140 issue pages? 10.1016/j.worlddev.2020.105357 PMC8446704 34548744

[CR10] Fraga, J., Robledo, D. 2022. Covid-19 and Sargassum blooms: impacts and social issues in a mass tourism destination (Mexican Caribbean). *Maritime Studies*. 10.1007/s40152-022-00267-0. 10.1007/s40152-022-00267-0 PMC9067340 35531368

[CR11] Godamunne, V., A.J. Abdeen and R. Siriwardane-de Zoysa 2022. Shored curfews: constructions of pandemic islandness in contemporary Sri Lanka [add] 10.1007/s40152-022-00262-5 PMC8907555 35299652

[CR12] Gupta J, Bavinck M, Ros-Tonen M (2021). COVID-19, poverty and inclusive development. World Development.

[CR13] Heazle M (2020). Assessing COVID-19’s “known unknowns”: Potential impacts on marine plastic pollution and fishing in the South China Sea. Maritime Studies.

[CR14] Heazle, M. 2021. Assessing COVID-19’s “known unknowns”: potential impacts on marine plastic pollution and fishing in the South China Sea. *Maritime Studies* 20: 459–474. 10.1007/s40152-021-00237-y. 10.1007/s40152-021-00237-y PMC8367392 35299597

[CR15] Khan A, Ahmed SM, Sarr C (2021). Nourishing nations during pandemics: Why prioritize fish diets and aquatic foods in Africa. Maritime Studies.

[CR16] Köpsel V, de Moura Kiipper G, Peck MA (2021). Stakeholder engagement vs. social distancing—how does the Covid-19 pandemic affect participatory research in EU marine science projects?. Maritime Studies.

[CR17] Lam ME (2021). Ethical reflections on the COVID-19 pandemic in the global seafood industry: Navigating diverse scales and contexts of marine values and identities. Maritime Studies.

[CR18] Lamers M, Student J (2021). Learning from COVID-19? An environmental mobilities and flows perspective on dynamic vulnerabilities in coastal tourism settings. Maritime Studies.

[CR19] Manlosa AO, Hornidge AK, Schlüter A (2021). A. Aquaculture-capture fisheries nexus under Covid-19: impacts, diversity, and social-ecological resilience. Maritime Studies.

[CR20] Marschke M, Vandergeest P, Havice E (2021). COVID-19, instability and migrant fish workers in Asia. Maritime Studies.

[CR21] Perillo, G.M.E., C.M. Botero, C.B. Milanes et al. 2021. Integrated coastal zone management in the context of COVID-19. Ocean & Coastal Management 21010.1016/j.ocecoaman.2021.105687 10.1016/j.ocecoaman.2021.105687 PMC8118659 34007124

[CR22] Pita, P., G.B. Ainsworth, B. Alba et al. 2021. First assessment of the impacts of the COVID-19 pandemic on global marine recreational fisheries, *Frontiers in Marine Science* 25 October 2021 10.3389/fmars.2021.735741

[CR23] Rammelt, C. 2021: The impact of COVID-19 on the eradication of poverty: an incorrect diagnosis, *Third World Quarterly*

[CR24] Sackey AD, Tchouangeup B, Lamptey BL (2021). Outlining the challenges of Covid-19 health crises in Africa’s maritime industry: The case of maritime operations in marine warranty surveying practice. Maritime Studies.

[CR25] Steenbergen, D., P.T. Neihapi, D. Koran et al. 2020*.* COVID-19 restrictions amidst cyclones and volcanoes: a rapid assessment of early impacts on livelihoods and food security in coastal communities in Vanuatu. Marine Policy 121 10.1016/j.marpol.2020.104199 PMC7487203 32952270

[CR26] van Tatenhove JPM (2021). COVID-19 and European maritime futures: Different pathways to deal with the pandemic. Maritime Studies.

[CR27] Viteri Mejía, C., Rodríguez, G., Tanner, M.K et al. 2020. Fishing during the “new normality”: social and economic changes in Galapagos small-scale fisheries due to the COVID-19 pandemic. *Maritime Studies*. 10.1007/s40152-022-00268-z. 10.1007/s40152-022-00268-z PMC9072759 35538937

